# Neuronal Activity and Its Role in Controlling Antioxidant Genes

**DOI:** 10.3390/ijms21061933

**Published:** 2020-03-12

**Authors:** Jing Qiu, Owen Dando, James A. Febery, Jill H. Fowler, Siddharthan Chandran, Giles E. Hardingham

**Affiliations:** 1 UK Dementia Research Institute, The Medical School, University of Edinburgh, Chancellor’s Building, Edinburgh EH16 4SB, UK; Jing.Qiu@ed.ac.uk (J.Q.); Owen.Dando@ed.ac.uk (O.D.); Siddharthan.Chandran@ed.ac.uk (S.C.); 2 Centre for Discovery Brain Sciences, University of Edinburgh, Hugh Robson Building, George Square, Edinburgh EH8 9XD, UK; james.febery@ed.ac.uk (J.A.F.); Jill.Fowler@ed.ac.uk (J.H.F.); 3 Centre for Clinical Brain Sciences, University of Edinburgh Chancellor’s Building, Edinburgh, EH16 4SB, UK

**Keywords:** neuroprotection, neurodegeneration, oxidative stress, signal transduction, synaptic activity, gene transcription, antioxidants, astrocytes

## Abstract

Forebrain neurons have relatively weak intrinsic antioxidant defenses compared to astrocytes, in part due to hypo-expression of Nrf2, an oxidative stress-induced master regulator of antioxidant and detoxification genes. Nevertheless, neurons do possess the capacity to auto-regulate their antioxidant defenses in response to electrical activity. Activity-dependent Ca^2+^ signals control the expression of several antioxidant genes, boosting redox buffering capacity, thus meeting the elevated antioxidant requirements associated with metabolically expensive electrical activity. These genes include examples which are reported Nrf2 target genes and yet are induced in a Nrf2-independent manner. Here we discuss the implications for Nrf2 hypofunction in neurons and the mechanisms underlying the Nrf2-independent induction of antioxidant genes by electrical activity. A significant proportion of Nrf2 target genes, defined as those genes controlled by Nrf2 in astrocytes, are regulated by activity-dependent Ca^2+^ signals in human stem cell-derived neurons. We propose that neurons interpret Ca^2+^ signals in a similar way to other cell types sense redox imbalance, to broadly induce antioxidant and detoxification genes.

## 1. The Nrf2 Pathway Is Weak in Neurons

When placed alone in the absence of supporting glial cells, forebrain neurons have a lower capacity than astrocytes to withstand oxidative insults., particularly those involving peroxide exposure or generation. The principle peroxide-detoxifying system in non-neuronal cells, the glutathione (GSH) system, has low capacity in neurons compared to astrocytes and neurons store far less glutathione than their glial neighbors [1,2]. As a result they rely on nearby glia for antioxidant support: in response to oxidative stress astrocytes release glutathione which is broken down and taken up by neurons for their own use [3,4,5,6,7]. Other glutathione-utilizing enzymes (specifically glutathione transferase M2-2) have also been reported to be secreted by glia for uptake by neurons with neuroprotective consequences [8].

In astrocytes, as with many cell types, glutathione steady state levels are maintained through a feedback inhibitory mechanism whereby GSH inhibits the rate determining step in GSH biosynthesis, catalyzed by Glutamate-cysteine ligase (GCL). However, glutathione biosynthetic capacity is further regulated at the transcriptional level by a clever system that (most cells) possess to homeostatically regulate antioxidant, detoxification and cytoprotective genes in response to metabolic challenge. This system is centered on the transcription factor Nrf2 [9,10,11], constitutively translated but ordinarily targeted for ubiquitin-mediated degradation by its inhibitor Keap1. However, in response to oxidative stress, one or more of Keap1′s redox-sensitive cysteine residues become oxidized, preventing Nrf2′s degradation, causing its accumulation and translocation to the nucleus [12,13]. Here, Nrf2 induces the expression of genes whose promoters contain its cognate binding site, referred to as the Antioxidant Response Element (ARE) [14]. Keap1 also possesses sensors for heavy metal stress, alkenals and nitric oxide, thus offering a variety of mechanisms by which cells sense stress and turn on a battery of ARE-containing genes [15,16]. In the brain, Nrf2 signaling is activated in astrocytes, contributing to ischemic preconditioning [16,17].

In contrast to astrocytes and most other cell types, forebrain neurons have a very weak Nrf2 pathway, due to expressing Nrf2 mRNA at very low levels, compared to astrocytes, in both rodent and human systems [3,18]. Moreover, what little Nrf2 is present in neurons is highly unstable [19]. As a result, any stresses that prevent Keap1-mediated degradation are ineffective simply because there is too little Nrf2 present in the first place [18]. Even genetic deletion of Keap1 has no effect on Nrf2 target genes in cortical neurons, unlike in other cell types such as astrocytes which exhibit a marked induction of basal expression of Nrf2 target genes. Examination of the relative level of expression of Nrf2 target genes reveals that they are much lower in neurons than astrocytes and that genetic deletion of Nrf2 lowers Nrf2 target gene expression in astrocytes, it has no effect in neurons [18]. Thus, Nrf2 contributes little to the basal expression of ARE-containing genes in neurons.

## 2. The Forebrain Neuronal Nrf2 Pathway Is Developmentally Shut off

The very low expression of Nrf2 in neurons compared to astrocytes is surprising given that both arise from the same population of neural progenitor cells. Interestingly, very young neurons do express quite high levels of Nrf2 and respond to Nrf2 activating stimuli with the accumulation of Nrf2 and induction of Nrf2 target genes [18]. However, as neurons mature, so levels of Nrf2 decline by a mechanism involving the epigenetic repression of the Nrf2 promoter leading to histone hypoacetylation around the Nrf2 transcription start site. The biological reason developing neurons should require the shut-off of the Nrf2 pathway is not fully understood, however clues have come from observing the effects of ectopically expressing Nrf2 in developing neurons at a time when it is ordinarily being repressed. While Nrf2 expression at this stage has the expected effect of enhancing antioxidant defenses and rendering neurons resistance to oxidative insults, it also had the effect of inhibiting development, with both dendritic outgrowth and arborization and synaptogenesis, severely retarded [18]. The basis of these effects by Nrf2 appears to be the repression of redox-sensitive signaling pathways critical for development, including the Wnt and c-Jun N-terminal kinase (JNK) pathways [18]. It remains to be seen whether Nrf2 pathway hypofunction is also a feature of other neuronal cell types, such as those in the cerebellum, striatum, midbrain and spinal cord, so the following sections apply primarily to cortical neurons.

## 3. Metabolic Adaption Induced by Neuronal Ca^2+^ Signals

Neuronal Nrf2 levels remain low in maturity, robbing them of an important stress response pathway. It could be argued that neurons in particular would benefit from a functional Nrf2 pathway: they are highly metabolically active, consuming large amounts of adenosine triphosphate (ATP) to maintain their resting membrane potential. Moreover, their primary source of energy is oxidative phosphorylation, a process that can lead to reactive oxygen species (ROS) production when electrons escape the transport chain. Importantly, this energy requirement and attendant ROS production, increases dramatically when neurons are electrically active [20,21]. Rather than simply maintaining ionic balance and membrane potential in the face of leak currents, the neuron must restore these balances after membrane depolarization, clear the synapse of neurotransmitter and replenish vesicle pools, all of which involve energy-requiring transport against concentration gradients. To meet these increased demands for energy, neurons do not just rely on laws of mass action: Ca^2+^ influx, triggered following membrane depolarization (through voltage-gated Ca^2+^ channels) or excitatory neurotransmitter release (e.g., through glutamate-gated N-Methyl D-Aspartate (NMDA) receptors) actively promote oxidative phosphorylation. Cytoplasmic Ca2+ - activates the Aralar component of the malate–aspartate shuttle (MAS) which functions to deliver reducing equivalents into the mitochondria and promotes pyruvate production [22,23]. Additionally, mitochondrial Ca^2+^ triggers the activation (and dephosphorylation) of pyruvate dehydrogenase, as well as activating several Ca^2+^ dependent enzymes within the tricarboxylic acid (TCA) cycle [24,25,26,27]. Thus, activity-dependent Ca^2+^ signals help the neuron to meet elevated energy demands, however, the side effect is increased ROS production [26,28,29,30,31,32], GSH utilization [33] and consequent need for enhanced antioxidant capacity.

## 4. Neuronal Regulation of Antioxidant Defenses

At face value, this coupling of enhanced ROS production to increased intrinsic antioxidant defenses would appear an ideal role for Nrf2 to play, however this mechanism is not available to neurons. Nevertheless, activity-dependent Ca^2+^ signals do lead to the transcriptional induction of antioxidant genes and consequently elevated capacity of both the glutathione and thioredoxin antioxidant systems [33,34,35]. For example, activity-dependent induction of catalytic (Gclc) and modifier (Gclm) subunits of glutamyl-cysteine ligase increase neuronal GSH biosynthesis capacity, boosting antioxidant defenses in vitro and in vivo. Indeed, reducing activity-dependent Ca^2+^ transients in vivo (by NMDAR antagonism) uncouples activity from Gclc expression, which leads to GSH depletion and neurodegeneration [33]. Interestingly, several of the antioxidant pathway genes induced by activity-dependent Ca^2+^ signals, such as Gclc, Gclm, Gsr, Srxn and Txnrd1 and Slc7a11 are known Nrf2 target genes, so how can neurons promote their expression if their Nrf2 pathway is not functional?

## 5. Neuronal Ca^2+^ Signals Can Induce Nrf2 Target Genes Independently of Nrf2

One fact often overlooked in the study of Nrf2 target genes is their potential to be controlled by transcription factors other than Nrf2. The transcription factor family AP-1 (Fos/Jun) is a known regulator of a number of known Nrf2 target genes, including Srxn1, Hmox1 and Nqo1. Interestingly, in these genes the Nrf2 binding ARE (typical consensus TMAnnRTGA(Y/G)nnnGCR) contains an embedded AP-1 site. For example the mouse Srxn1 ARE sequence is TCACCCTGAGTCAGCG and the mouse Hmox1 ARE sequence is TCTGCTGAGTCAAGGTCCG (the AP-1 consensus sequences are underlined) [36,37]. Several studies have shown that AP-1 can contribute to the basal expression of ARE-containing genes, which can be further induced by Nrf2-activating stimuli [36]. Of course, most signal responsive promoters contain multiple elements, offering further scope for Nrf2-independent control of “Nrf2 target genes”. A well-studied example is the ARE-containing gene Slc7a11, which encodes the cystine/glutamate antiporter xCT. The Slc7a11 promoter also contains binding sites for the transcription factor ATF-4, offering an alternative route to its transcriptional regulation [38]. Given this, there is scope for Nrf2 target genes to be induced in a Nrf2-independent manner. This has been confirmed in the case of two examples: activity-dependent induction of mouse Srxn1 is Nrf2-independent [39] and AP-1 dependent [40], while activity-dependent induction of mouse Slc7a11 is Nrf2-independent and is ATF-4 dependent [41].

**Figure 1:**
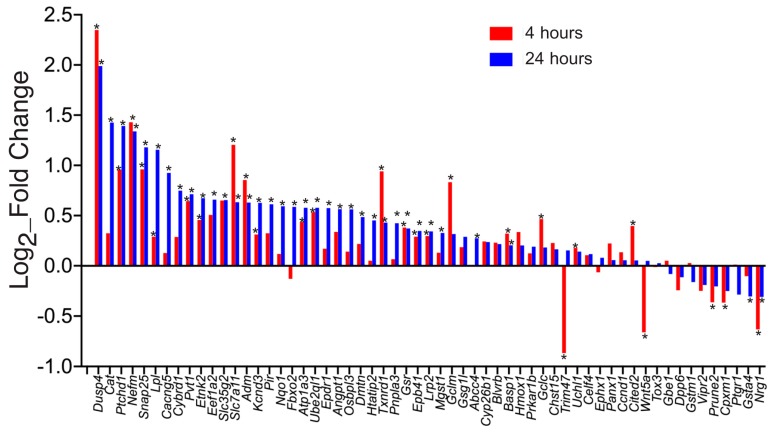
Log_2_ fold change of the indicated Nrf2-regulated genes in human embryonic stem cell (ESC)-derived cortical neurons stimulated with high K^+^ for the indicated period of time. The data come from a meta-analysis of that published previously [42]. * *p* <0.05 (*n* = 3).

Thus, it is clear that certain Nrf2 target genes can be regulated by activity-dependent Ca^2+^ signals despite not having a functional Nrf2 pathway. However, it does not tell us what proportion of Nrf2 target genes can be controlled in this way by electrical activity. To gain an impression of this, we interrogated our previously reported RNA-seq data set of activity-dependent gene expression in human embryonic stem cell-derived neurons [42]. In this study we generated dissociated glutamatergic cortical-patterned neurons from human embryonic stem cells (hESC^CORT^-neurons) [43,44] and studied transcriptional responses to Ca^2+^ influx through l-type Ca^2+^ channel activation, achieved by KCl-induced membrane depolarization in the presence of the l-type Ca^2+^ channel agonist FPL64176 (KCl/FPL). Two timepoints were studied: 4 h and 24 h. We then cross-referenced this data set against a set of Nrf2-regulated genes, curated from an RNA-seq data set of astrocytes sorted from transgenic mice which overexpress Nrf2 specifically in astrocytes [45] (GFAP-Nrf2). 59 genes are induced >2-fold in GFAP-Nrf2 astrocytes (relative to wild-type astrocytes) that were expressed > 1FPKM on average in the hESC^CORT^-neurons (data not shown). Of these 59 genes, 39 genes were found to be significantly induced in hESC^CORT^-neurons (*p* < 0.05) at either 4 h or 24 h timepoints, including *GCLC*, *GCLM*, *GSR*, *NQO1*, *TXNRD1 CAT* and *SLC7A11* and only 6 genes were significantly down-regulated (Figure 1).

Collectively this illustrates that a substantial proportion of Nrf2-regulated genes can be controlled by Ca^2+^ signals in human neurons. It makes teleological sense to mount an adaptive/protective response to Ca^2+^ signals, since substantial or repetitive Ca^2+^ influx into neurons places metabolic demands on neurons which may be unsustainable or injurious in the absence of adaption [46,47,48,49]. This type of metabolic homeostatic control regulated by Ca^2+^ signals draws parallels with other Ca^2+^ -dependent rebalancing processes in neurons, such as homeostatic plasticity of synaptic and intrinsic properties [50,51,52]. That said, Ca^2+^ signals control several programs of cytoprotective gene expression that Nrf2 is not implicated in, such as the repression of apoptotic genes and FOXO-dependent gene expression [53,54], the induction of nuclear calcium and CREB-dependent pro-survival genes [55,56,57,58] and the manipulation of TrkB signaling [59]. Moreover, it is important to note that all the aforementioned effects of Ca^2+^ signals are in response to non-toxic, physiological levels of Ca^2+^, excessive Ca^2+^ influx, particularly through extrasynaptic NMDA receptors, induces a very different gene expression profile which has a net toxic, rather than protective effect [60,61,62].

## 6. Concluding Remarks

It remains to be seen whether Ca^2+^ signals regulate Nrf2 target genes in other excitable cells, such as those of smooth and skeletal muscle or cell types where Ca^2+^ signals herald major metabolic or developmental changes, such as oocytes and T-cells. Nevertheless, the overlap between the neuronal activity-dependent transcriptome and the Nrf2-regulated transcriptome suggest that neurons interpret Ca^2+^ signals in a similar way to other cell types sense oxidative stress, to broadly induce antioxidant and cytoprotective genes to confer greater cellular resilience in the face of greater metabolic demand.
